# Interactions between color and gloss in iridescent camouflage

**DOI:** 10.1093/beheco/arad050

**Published:** 2023-06-14

**Authors:** Dylan H N Thomas, Karin Kjernsmo, Nicholas E Scott-Samuel, Heather M Whitney, Innes C Cuthill

**Affiliations:** School of Biological Sciences, University of Bristol, 24 Tyndall Avenue, Bristol BS8 1TQ, UK; School of Biological Sciences, University of Bristol, 24 Tyndall Avenue, Bristol BS8 1TQ, UK; School of Psychological Science, University of Bristol, 12a Priory Road, Bristol BS8 1TU, UK; School of Biological Sciences, University of Bristol, 24 Tyndall Avenue, Bristol BS8 1TQ, UK; School of Biological Sciences, University of Bristol, 24 Tyndall Avenue, Bristol BS8 1TQ, UK

**Keywords:** camouflage, defensive coloration, iridescence, specular reflectance, gloss

## Abstract

Iridescence is a taxonomically widespread form of structural coloration that produces often intense hues that change with the angle of viewing. Its role as a signal has been investigated in multiple species, but recently, and counter-intuitively, it has been shown that it can function as camouflage. However, the property of iridescence that reduces detectability is, as yet, unclear. As viewing angle changes, iridescent objects change not only in hue but also in intensity, and many iridescent animals are also shiny or glossy; these “specular reflections,” both from the target and background, have been implicated in crypsis. Here, we present a field experiment with natural avian predators that separate the relative contributions of color and gloss to the “survival” of iridescent and non-iridescent beetle-like targets. Consistent with previous research, we found that iridescent coloration, and high gloss of the leaves on which targets were placed, enhance survival. However, glossy targets survived less well than matt. We interpret the results in terms of signal-to-noise ratio: specular reflections from the background reduce detectability by increasing visual noise. While a specular reflection from the target attracts attention, a changeable color reduces the signal because, we suggest, normally, the color of an object is a stable feature for detection and identification.

## Teaser

The shininess of iridescent beetles makes them more conspicuous, but their changeable hues reduce their detectability by bird predators. We showed this by measuring predation rates on artificial beetle-like prey in the field. We found that the variable color of iridescent prey enhanced survival, but glossy targets survived less well than matt. Although gloss increases salience, iridescence reduces detectability because the color of an object is normally a stable feature for detection and identification.

## INTRODUCTION

Iridescence is a taxonomically widespread form of structural coloration that tends to be characterized by bright, vibrant hues that are—their defining feature—angle-dependent (Vigneron et al. 2006; Doucet and Meadows 2009; White 2018). Although there are three main structural mechanisms that can produce iridescent coloration (Kinoshita 2008; Seago et al. 2009), by far the most common and best understood in nature is the multilayer reflector (Seago et al. 2009), which is responsible for the iridescence seen in many beetles (Seago et al. 2009; Yoshioka et al. 2012) and butterflies (Vukusic et al. 2001). Because iridescence is so prevalent, it has many hypothesized biological functions (Meadows et al. 2009), including species recognition (e.g., Rutowski 1977), age identification (e.g., Papke et al. 2007), sex identification (e.g., Lim et al. 2007), mate choice (e.g., Kemp 2008), a by-product of non-visual roles in thermoregulation (e.g., Heilman and Miaoulis 1994; Biró et al. 2003) and, in plants, a mechanism for enhancing light capture (Jacobs et al. 2016). Changeable intense coloration would seem an unlikely candidate for camouflage, but recent evidence suggests that iridescence can conceal, particularly when the target is on a glossy background (Kjernsmo et al. 2020). All Kjernsmo et al.’s artificial prey were glossy, so it is unclear the extent to which the beneficial effects of iridescence are dependent upon this surface property. As we explain below, Franklin and Ospina-Rozo (2021) proposed that a glossy surface could reduce detectability independent of iridescence. We replicate Kjernsmo et al.’s (2020) experiment but independently manipulate the glossiness of prey to determine the effects of gloss per se and in interaction with iridescence. We also included background gloss as a predictor because it has previously been shown to significantly affect target survival (Kjernsmo et al. 2020).

A camouflage function for iridescence was first suggested by Abbott Thayer in the early 20th century (Thayer 1909). He proposed that iridescence allows animals to “appear ‘dissolved’ into many depths and distances,” describing it as “one of the prime factors of disguise” (Thayer 1909, p.87). According to Thayer’s statements, both background matching—in which animals have colors and patterns that match those of their background (Merilaita and Stevens 2011)—and disruptive coloration—in which contrasting colors interrupt the true outline while simultaneously generating false edges (Cott 1940; Cuthill et al. 2005; Stevens and Cuthill 2006; Stevens and Merilaita 2009b)—may be important. Until recently, Thayer’s ideas lacked empirical support, but Kjernsmo et al. (2020) showed that iridescent targets, made from real jewel beetle (*Sternocera aequisignata*) elytra, were less detectable to birds and humans than non-iridescent targets, particularly when viewed against glossy leaves. However, exactly how iridescence provides camouflage remains unclear. Thayer (1909) believed that iridescent surfaces achieve camouflage through their color. Surface specularity could also be important. Franklin and Ospina-Rozo (2021) suggested that specular reflectance (from glossy surfaces) could aid in camouflage; this might have important implications for iridescence as camouflage.

Specularity is the proportion of light reflected at the mirror angle (mirror-like reflection of light, where the angle of reflection equals the angle of incidence), as contrasted with diffuse reflection, where incident light is scattered in all directions. Because smooth objects produce specular reflections, it is an important contributor to the perception of a surface as glossy (Chadwick and Kentridge 2015; Franklin and Ospina-Rozo 2021). For example, a glossy material will have bright white highlights across its surface, in addition to whatever the base color might be: these are specular reflections. It has been suggested that these highlights could constitute high-contrast patches that create edge disruption (Franklin and Ospina-Rozo 2021). This could arise through lateral inhibition (strong signals masking the weak signal of the true outline; Stevens et al. 2009; Merilaita et al. 2017) or distraction from the true outline (Dimitrova et al. 2009). In either case, such edge disruption by glossy signals could contribute to concealment through disruptive coloration. Gloss could also theoretically improve camouflage through background matching if an animal is viewed atop a background with which it shares a similar level of glossiness. Because both iridescence and specular reflections produce an alteration in the light reflected from an object as the angle of viewing changes, they create instability in the visual signal (Stuart-Fox et al. 2021). This signal instability potentially reduces the signal-to-noise ratio (SNR), the key determinant of effective crypsis (Merilaita et al. 2017; Galloway et al. 2020). In the case of gloss, there is recent evidence that background gloss can conceal targets regardless of their own glossiness, presumably because of the generation of background noise (Kjernsmo et al. 2020). Based on the above observations, one could hypothesize that a combination of iridescent coloration and gloss (atop a glossy background) would achieve the most effective camouflage.

In this investigation, we monitored the “survival” of artificial beetle-like targets under natural avian predation. The aim of our research was to start exploring the mechanisms behind iridescence as camouflage, with a particular focus on separating the relative contributions of target color and gloss. We were interested in specular gloss, and any mention of gloss herein refers to that involving specular reflection. We predicted that iridescent coloration would provide camouflage irrespective of target glossiness but that (for the reasons outlined above) the camouflaging effect would be stronger for glossy than matt targets.

## METHODS

We followed a similar experimental procedure to Kjernsmo et al. (2020), with modifications to allow us to investigate the effects of both target color and gloss (Kjernsmo et al. 2022), and any possible interactions with background gloss, on survival. The experiment followed a 2 × 5 factorial design, with all possible combinations of target color (iridescent, green, blue, black, and what Kjernsmo et al. termed a “static rainbow”; see below) and target gloss (glossy, matt). Natural variation in background gloss was recorded as an unmanipulated covariate.

### Target production

**Figure 1:**
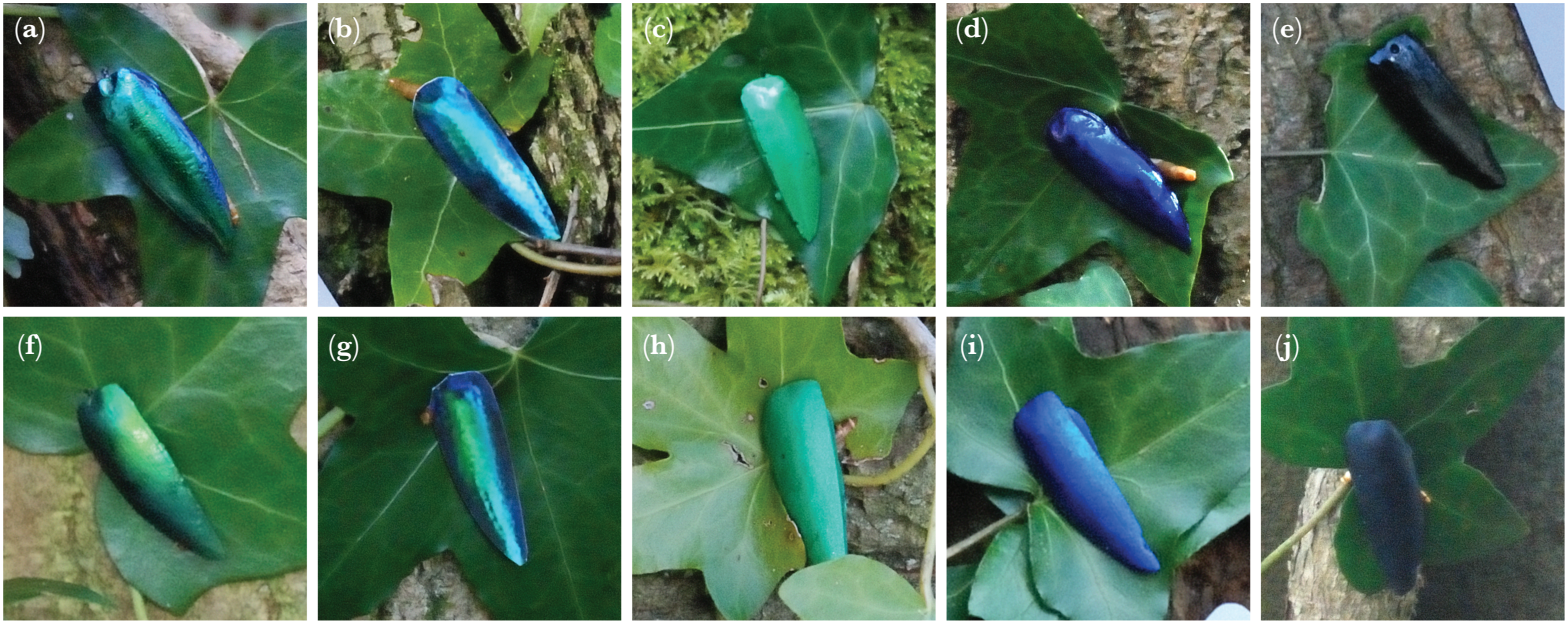
Artificial beetle-like targets pinned to English ivy (*Hedera helix*) leaves. From left to right, color treatments are: iridescent; static rainbow; green; blue; black. Targets in images a–e have a glossy surface appearance; targets in images f–j have a matt surface appearance.

Targets were produced using a mixture of real and artificial elytra of an Asiatic jewel beetle (*Sternocera aequisignata*; Figure 1). Real elytra were used for the iridescent targets; the four non-iridescent control targets were based on molds of real elytra. Green, blue, and black targets were created from 2-Ton Epoxy resin (Devcon, ITW Performance Polymers, Shannon, County Clare, Ireland). A 50:50 mixture of resin and hardener (6.5 g each) was mixed with 150 mg of black pigment (L. Cornelissen & Son, London, UK) and poured into elytra-shaped molds. These molds had been created by gently pressing real elytra into Elite HD+ Light Body silicone dental putty (Zhermack, Badia Polesine, Italy) to produce negative impressions. Several elytra were used to create a variety of differently sized targets, accommodating the morphological variation seen in wild beetle populations. Nail varnishes were used to paint the artificial targets, as selected by Kjernsmo et al. (2020) to match the reflectance peaks of the main colors observed when viewing the iridescent target at different angles. We did not measure reflectance spectra ourselves, instead relying on the fact that we used the same varnishes and target creation methods as Kjernsmo et al. (2020). Green targets were painted with two nail varnishes (“163 Metallic Green,” Kleancolor, Santa Fe Springs, USA; “Peacock Green,” N°7, The Boots Company PLC, UK), mixed in a 50:50 ratio. Blue and black targets were each painted with a single nail varnish (“661 Ocean Blue” (Maybelline, New York, USA) and “Blackjack2” (Collection, LF Beauty, UK), respectively). All three targets received two coats of nail varnish, one to produce the desired color, and one to remove UV reflections, as well as a third type of varnish to manipulate glossiness (see below). Although Kjernsmo et al. (2020) created a purple target, purple is only seen in the real beetle from extreme viewing angles, and—in addition—the difference in survival between this and the blue target was relatively small, so we omitted the purple target from this study. We created “static rainbow” (SR) targets to separate the effect of multiple colors per se from the angular change in color exhibited by iridescence. To create the SR targets, a selection of real beetle elytra were photographed from directly above under natural lighting using a Nikon D90 DSLR camera (Nikon Corporation, Tokyo, Japan). An X-Rite ColorChecker Passport (X-Rite, Grand Rapids, Michigan, USA) was included in each photograph so that they could be calibrated following Stevens et al. (2007). Photographs were printed onto photographic paper (Epson Premium Glossy Photo Paper S042155), using an Epson SureColor SC-P600 (Seiko Epson Corporation, Suwa, Nagano, Japan), ensuring that the size of the printed beetles matched the size of the real beetles. Since we photographed unmanipulated elytra, the SR targets retained the spatial gloss pattern (as it appears from the angle of the photography) of the real elytra. However, the spectral highlights (white patches) lacked the intensity of true gloss. The printed beetles were carefully cut and then shaped around the bulb of a plastic Pasteur pipette to give a slightly rounded appearance akin to the real beetles.

To enable us to pin targets to vegetation in the field experiments, drawing pins were stuck to the backs of all targets. For the green, blue, and black targets, these were embedded into the target itself while the resin was still malleable; for the iridescent and SR targets, these were stuck using a few small drops of 2-Ton Epoxy resin (50:50 mix of resin and hardener, with added pigment, as before). All targets were given a single layer of transparent nail varnish (“SuperStay 3D Gel Effect,” Maybelline, New York, USA) to control for any differences in olfactory cues and to eliminate ultraviolet reflectance. The latter allowed the estimation of avian-perceived colors without the need for UV photography. Finally, targets were sprayed with three layers of one of two types of fixative (Professional Gloss Varnish/Professional Matt Varnish, Windsor & Newton, London) so that half the targets had a glossy and half a matt surface appearance (Kjernsmo et al. 2022; see Supplementary Figure S1).

### Field experiment

The experiment had a randomized block design, with each of the 10 experimental blocks conducted in a different area of Leigh Woods National Nature Reserve, North Somerset, UK (51°27ʹ20.7ʹʹN 2°38ʹ28.9ʹʹW) between March and April 2020. Within each block, consisting of a ca. 1 km path, 10 replicates of each of the 10 treatments were randomly assigned to substrates. The beetle-like targets were pinned on English ivy (*Hedera helix*) leaves against tree trunks, roots, or plant shoots (Figure 1), and their survival over a 96 h period was monitored. Although Kjernsmo et al. (2020) pinned their targets to various plant species, of which ivy was the commonest, we chose ivy because it shows substantial variation in glossiness (see Supplementary Figure S1) while allowing us to eliminate possible plant-species effects on predation rates. Another reason to choose ivy is that it grows on trees, allowing secure attachment of each target, through an ivy leaf, to the root or trunk below. Some leaves had to be moved slightly to do this, but all were still alive and attached to their (ivy) plant. The artificial prey consisted of two parts: an inedible elytron and an edible frozen-then-thawed mealworm (*Tenebrio molitor*), which was pinned beneath the elytron. After picking a suitable leaf between ground level and a height of ~2 m, a target was selected at random by drawing it blindly from a well-mixed bag, and pinned through the leaf into the wood on which the ivy was growing. The mealworm was partially visible, with about ~2 mm protruding from beneath the elytron. After pinning, we checked the targets at 24, 48, 72, and 96 h for signs of avian predation. Avian predation was inferred from the partial or full consumption of the mealworm (and provided that the inedible elytron was still pinned to the leaf). If a target had been dislodged, or showed signs of spider or slug predation (hollow exoskeleton or slime trails, respectively), it was considered predated-but-censored (see below). It is possible that we misclassified predation events by ants as avian, because ants can easily remove such prey within 24 h, but as ants would largely hunt using olfactory cues, this would add noise to the data rather than produce a bias. Predated targets were collected when found, along with the leaves they had been pinned to. Any remaining targets after 96 h were also collected (along with their leaves). When the leaves were collected, they were labeled, using a permanent black marker pen, with a code that corresponded to the target, and placed in a plastic bag for subsequent measurement. Since both iridescence (Stuart-Fox et al. 2021) and gloss (Chadwick and Kentridge 2015) vary depending on lighting, we also noted the weather conditions throughout the investigation. Lighting was consistent throughout the experimental period. The weather was mostly sunny and dry (about 60% of experimental days), the remainder cloudy and dry and, since all targets were pinned to leaves within a woodland environment, most would have been subjected to similar lighting conditions: the diffuse illumination of forest shade (Endler 1993).

### Gloss measurements and color analysis

Leaf gloss measurements were made on the day of collection using GlossTools v2.1 and a ZGM 1120.268 glossmeter (Zehntner Testing Instruments, Sissach, Switzerland). Although this glossmeter measures specular light reflectance at three angles (20°, 60°, and 85°), measurements were only taken from the 60° angle because this is particularly suitable for surfaces of small size (Whitney et al. 2012). Five measurements were taken from each leaf (avoiding the easily visible veins), and the average was used. Leaves were kept completely flat when taking measurements. The glossmeter was calibrated using the supplied black polished glass standard (refractive index 1.567) before any measurements were made, and the units we present are gloss as a percentage of that standard. Gloss measurements were also taken from a subsample of 10 beetle targets per treatment, to validate the subjective impression that the varnishes had changed gloss as intended. Because our beetle targets were not flat, thus adding measurement error, we took five measurements from different points along the sagittal plane. These measurements allowed us to assess the differences between the two gloss categories (see Supplementary Figure S1).

### Statistical analyses

All statistical analyses were performed using R (v. 4.0.0; R Core Team 2020). We used mixed-effects Cox regression, using the “coxme” package (Therneau 2020a), to assess the manipulated fixed effects of a priori interest, target color (five levels), and target gloss (two levels). We also fitted candidate models with the unmanipulated natural variation in the gloss of each leaf on which targets were placed (a continuous variable) because we considered interactions with the experimentally manipulated factors plausible. Kjernsmo et al. (2020) had found that the survival of iridescent, but not non-iridescent, targets, was greater on glossy leaves (a color-by-leaf-gloss interaction). We also predicted that glossy targets would have higher salience, and so lower survival, on less glossy leaves (a target-gloss-by-leaf-gloss interaction). Block and target identity (as targets were reused in multiple blocks) were included in all candidate models as random effects. All possible models up and including the three-way interaction between target color, target gloss, and leaf gloss were fitted and compared using Akaike’s Information Criterion to identify the model(s) most consistent with the data. Models within two units of the model with the lowest AIC value were also considered plausible (Burnham and Anderson 2002). Post-hoc Tukey contrasts comparing the different target color treatments were performed using the “multicomp” package (Hothorn et al. 2008). The “png” package was used for reading in PNG images for plot creation (Urbanek 2013).

## RESULTS

Across the whole experiment, 73.4% of beetle targets showed evidence of avian predation; the remaining 26.6% of targets were classed as censored in the survival analysis, including 48 that survived until the end of the 96 h experimental period, with the rest eaten by slugs or spiders, dislodged, or lost. The repeatability (intra-class correlation coefficient) of leaf gloss measurements was 0.84, indicating that leaf gloss could be measured reliably. We also note that there was comparable variation in background glossiness in our study to that in Kjernsmo et al. (2020; see Supplementary Figure S1). To compare multiple models, the datasets must be the same for each. As such, we excluded a small percentage of the cases (0.3%) where data were missing on leaf gloss.

**Figure 2:**
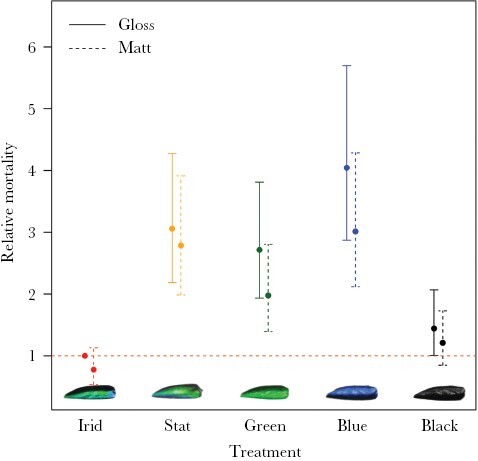
Odds ratios (±95% confidence intervals) showing relative mortality of five color treatments (iridescent; static rainbow; green; blue; black) under avian predation. Odds ratios were obtained from a mixed-effects Cox regression model. All color treatments are compared to the glossy iridescent treatment. Glossy targets are indicated by solid 95% confidence interval lines; matt targets are indicated by dashed interval lines.

The model with the lowest AIC value contained the main effects of target color (five levels), target gloss (two levels), and leaf gloss (continuous), plus the target-gloss-by-leaf-gloss interaction (Table 1). Only one other model fell within 2 AIC units of this best model: one with (only) the three main effects. Before considering the interaction between gloss of the target and that of the leaf, we note that we have no evidence for target color interacting with other factors. In both of the “best” models, pair-wise comparisons of the colors showed that the iridescent targets survived significantly better than each of the four other colors (Figure 2, Table 2 shows the results from the model that includes the target-gloss-by-leaf-gloss interaction; the results from the model with only main effects is virtually identical). Of the other treatments, black survived significantly better than blue, green, or static rainbow, and green survived better than blue (Table 2). The survival can be summarized as iridescent > black > green = static rainbow > = blue, where green survived significantly better than blue, although there was no significant difference in survival between static rainbow and blue.

**Table 1:** Log-likelihoods, degrees of freedom, and Akaike’s Information Criteria (AIC) of multiple mixed-effects Cox regression models for survival analysis of beetle-like targets under avian predation

Fixed effects	logLik	df	AIC	ΔAIC
C + G + L + C:G + C:L + G:L + C:G:L	−4299.74	26.17	8651.81	17.66
C + G + L + C:G + C:L + G:L	−4300.87	22.18	8646.09	11.94
C + G + L + C:G + C:L	−4302.91	21.15	8648.13	13.97
C + G + L + C:G + G:L	−4302.20	18.17	8640.74	6.59
C + G + L + C:L + G:L	−4301.66	18.20	8639.71	5.55
C + G + L + C:L	−4303.55	17.18	8641.46	7.30
C + G + L + C:G	−4304.18	17.14	8642.65	8.50
**C + G + L + G:L**	−**4301.04**	**16.04**	**8634.15**	**0.00**
**C + G + L**	−**4304.82**	**13.17**	**8635.99**	**1.83**
C + G	−4311.09	12.19	8646.56	12.41
C + L	−4303.31	17.91	8642.43	8.28
G + L	−4271.00	93.18	8728.36	94.20
C	−4314.84	11.16	8652.00	17.85
G	−4281.97	93.60	8751.14	116.99
L	−4268.95	96.12	8730.16	96.00
Intercept only	−4280.42	95.70	8752.25	118.10

All models contained Block and Target identity as random effects. All possible candidate models for fixed effects of target color (C), target gloss (G) and leaf gloss (L) were tested. The last column, ΔAIC, shows the difference between the best model (lowest AIC) and each model. The two models that the data are most consistent with are highlighted in bold.

**Table 2:** Pairwise comparisons of color treatments to each other (iridescent, static rainbow; green; blue; black) in the best-fitting model from Table 1 (C+G+L+G:L), using Tukey contrasts

	Iridescent	Static	Green	Blue	Black
Iridescent	–	<0.001	<0.001	<0.001	0.017
Static	9.35	–	0.276	0.411	<0.001
Green	7.49	−1.98	–	0.003	<0.001
Blue	10.47	1.74	3.62	–	<0.001
Black	3.10	−6.51	−4.57	−7.86	–

Iridescent targets survived significantly better than the other colors. *P* values are presented in the upper right-hand corner; z scores are presented in the lower left-hand corner. The z scores have been calculated by subtracting the survival of the row color from that of the column color, such that a positive z-score indicates that the column survived better than the row treatment in the pairwise comparison.

**Figure 3:**
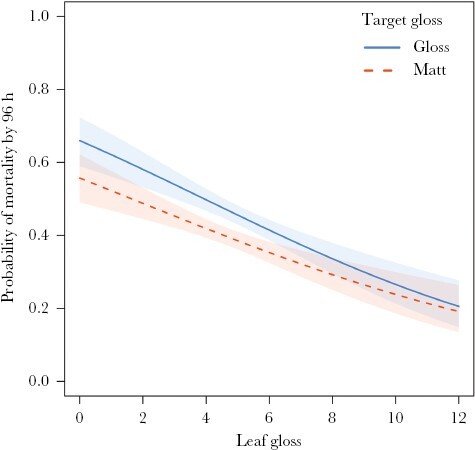
The probability of mortality by 96 h for glossy (solid blue line) and matt (dashed red line) targets on leaves of increasing glossiness (measured in gloss units), under avian predation. Estimates were obtained from a mixed-effects Cox regression model using the predict.coxme function from the coxme package; shaded regions span the 95% confidence intervals for each line.

If we consider the model with only main effects to be more parsimonious than the model with a gloss interaction, then this showed that matt targets experienced ca 20% lower predation rates than gloss (odds ratio = 0.80, 95% CI 0.69–0.93) and all targets had just under 10% lower predation on glossier leaves (odds ratio = 0.92, 95% CI 0.87–0.96). However, the model with a target-gloss-by-leaf-gloss interaction had a marginally lower AIC, so the data are also consistent with this slightly more complex model. The nature of the interaction was that glossy targets had higher mortality than matt targets on most leaves, but the difference disappeared on the glossiest leaves (Figure 3; odds ratio = 1.10, 95% CI 1.00–1.20). Note that the 95% CI for the interaction (just) includes 1, equivalent in a null hypothesis testing framework to no significant effect.

## DISCUSSION

In line with Kjernsmo et al. (2020), our results show that color changeability is the most important aspect of iridescence as camouflage, with iridescent targets surviving significantly better than all other treatments (Figure 2). We also expanded on the work of Kjernsmo et al. (2020) to investigate the importance of gloss in camouflaging iridescence. Contrary to Franklin and Ospina-Rozo’s (2021) suggestion that specular reflections might have a disruptive or distractive effect, matt targets survived significantly better than glossy targets. The effect of substrate gloss was in the opposite direction, with all targets surviving significantly better on glossier leaves than on less-glossy leaves. In a broader sense, the results of this investigation contribute to a widening literature on camouflaging iridescence. Our data support conclusions from previous research that have shown that iridescence can function as an effective form of defensive coloration (Fabricant et al. 2014; Pike 2015; Kjernsmo et al. 2018, 2020, 2022).

We cannot say whether iridescence is more effective than a “traditional” background-matching camouflage because the green of our (and Kjernsmo et al. 2020’s) targets was designed to match the dominant jewel beetle color, not ivy leaf green, and the greens of targets and leaves were different (see Supplementary Material). That said, a role for background matching has been suggested before (Schultz 1986; Seago et al. 2009) and might still be relevant: many iridescent insects, including the *Sternocera aequisignata* jewel beetle elytra we used in this study, appear green from a range of angles (Sun et al. 2013). Furthermore, although the ivy and target greens occupy different areas of color space (Supplementary Material), we extracted color and luminance measurements from single photographs in plain view, which do not fully characterize the iridescence—the angle of photography will have influenced the color values obtained. Therefore, at some viewing angles, there may be some degree of background matching or, because at lower viewing angles, the jewel beetles appear dark, at least lower salience. The latter would be aided by the fact that, although jewel beetle green is a poor match to ivy leaves, a variety of greens are common background colors in woodland, whereas blue is not. Background matching here might be generalized (Merilaita et al. 2001). In its native habitat, the green hues in the *Sternocera aequisignata* elytra might match the background colors more closely.

Alternatively, or in addition, iridescent camouflage might involve disruptive coloration. To achieve effective disruptive coloration, some degree of background matching is desirable, where some color patches blend with the background (Cott 1940; Stevens et al. 2006; Fraser et al. 2007; Stevens and Merilaita 2009a, b; Cuthill 2019). Although our targets did not completely match the colors of the ivy leaves (Supplementary Material), there may be sufficient generalized matching. The color patches on iridescent elytra that contrast with the green could then act to disrupt the outline or surface. This, combined with the color-shifting nature of iridescent surfaces, could create some sort of “dynamic” disruptive coloration that generates the appearance of different shapes as the viewer moves relative to the target, as suggested by Kjernsmo et al. (2020). Kjernsmo et al. (2018) demonstrated that multilayer iridescence interferes with object recognition via shape in bumblebees (*Bombus terrestris*), suggesting that iridescent camouflage could indeed act through disruptive coloration. Somewhat similarly, Pike (2015) found that iridescence interferes with the targeting of moving targets in Japanese quail (*Coturnix japonica*), concluding that this is the result of color changeability, making it harder to track iridescent surfaces. While there may be some disruptive coloration, this actually better illustrates how iridescence might interfere with perception through the generation of noise.

For camouflage to be successful, it must act to reduce the signal-to-noise ratio or SNR (Merilaita et al. 2017; Galloway et al. 2020). Although iridescence is usually associated with display, for it to function effectively as a signal (in both the evolutionary and information-theoretic sense), it must be combined with structures and/or behaviors that enhance its detectability and reliability (Stuart-Fox et al. 2021). Without such detectability-enhancing mechanisms, iridescent signals are unstable and so not reliably correlated with the property the receiver is attempting to predict (in the sexual selection context, mate quality, but in the predator–prey context of our study, object identity and thus edibility). Iridescent colors tend to be bright and assumed to be salient (Doucet and Meadows 2009), yet signal instability might make it difficult for animals to resolve shape information as well as determining color itself; or make it easier for useful color cues to be missed. As such, we interpret iridescence as camouflage from a SNR perspective and suggest that rather than forcing iridescent camouflage into one “traditional” category of camouflage, it is best to consider how, through color changeability, iridescent coloration might reduce the SNR sufficiently to enable concealment.

Changeable iridescent colors also have important implications for prey encounters. In the case of a foraging animal hunting static prey, when an iridescent surface is noticed, perhaps in peripheral vision, the animal may be initially attracted to the hue that is detectable at their current viewing angle. But as they move closer to investigate, the viewing angle and thus the perceived hue changes, meaning the surface no longer appears as before, or is even temporarily undetectable. Furthermore, given that many predators are themselves under predation risk and are thus constantly alert to potential threats (Lima and Dill 1990), a simple predator-checking saccade away from prey may be all that is necessary for an iridescent target to “disappear” and be hard to locate following an initial detection (Smart et al. 2020). Another effect of color changeability on predators could be that the changeable iridescent surfaces prevent search image formation. Search images are short-term perceptual filters that predators develop through frequent prey encounters and subsequent learning that enable fast identification of specific prey features (Tinbergen 1960; Pietrewicz and Kamil 1979; Langley 1996; Troscianko et al. 2018) and, in avian predators, learning of prey type seems to be heavily based on color (Kazemi et al. 2014; Gamberale-Stille et al. 2018; Lawrence and Noonan 2018; Corral‐Lopez et al. 2020). Because iridescent surfaces are unstable in their color signal, they might interfere with learning, such that search image formation is not possible. That said, recent experiments have shown that European honey bees (*Apis mellifera*) can accurately use chromatic cues to discriminate between iridescent and silver discs (Ng et al. 2022). This suggests that some species may indeed be able to learn to identify iridescent signals.

Interestingly, although iridescent targets survived significantly better than black targets, black still seemed to survive well. This is perhaps surprising in terms of their difference from ivy leaves (see Supplementary Material), but was a result also obtained by Kjernsmo et al. (2020). Maybe black targets were misclassified by predators as shadows, holes (whether whole organisms can be mistaken for holes has never been tested, but smaller hole-resembling patches have been shown to have camouflage benefits; Costello et al. 2020), feces, unpalatable slugs, or diseased leaves, and so tended to go unnoticed or be ignored. In the case of Kjernsmo et al.’s (2020) second experiment, involving humans searching for targets rather than birds, active avoidance can be discounted, but whether the success of black results from failed detection or misclassification deserves further investigation.

Contrary to predictions made by Franklin and Ospina-Rozo (2021)—that a reflective glossy surface could have a prominent role in camouflage through edge disruption or interference with search image formation—we found that matt targets survived significantly better than glossy targets. Initially, this may seem surprising, especially when considering that if glossy signals are noisy, then they should shift the SNR in favor of concealment. However, previous research may help to explain the camouflage-breaking costs of gloss seen in our study. The military has long recognized the advantages, when painting vehicles, of “dull mat finishes in all camouflage painting; to reduce the specular reflection of light on the surface” (US Company of Engineers 1941). Gloss is spectrally flat, and its specular reflections are of high intensity, so the bright white light will be highly salient against dark green vegetation unless that vegetation itself is glossy (or, as suggested by Kjernsmo et al. 2020, the surfaces are wet from rainfall). It has also been suggested that gloss can increase the salience of three-dimensional (3D) body cues (Adams and Elder 2014; Chadwick and Kentridge 2015; Nityanda and Read 2017; Adams et al. 2019). When the target and/or the observer changes position relative to the other, the position of specular highlights across a glossy surface shifts, following the contours of the body, potentially revealing the 3D shape (Chadwick and Kentridge 2015). Since 3D cues are important in visual search (Penacchio et al. 2015; Cuthill et al. 2016; Penacchio et al. 2018), this might facilitate detection and recognition, at least for monocular viewing, when stereopsis cues from binocular disparity are not available. Nityananda and Read (2017) and Adams et al. (2019) have suggested that specular highlights because they give different depth information to the two eyes from that of the rest of the surface, might interfere with the efficiency with which stereopsis breaks camouflage. We would assume that the avian predators in our study were largely using monocular search (Martin 2009), so whether gloss aids or impedes the visual search for binocularly searching predators, such as humans, deserves further investigation.

Currently, few studies have thoroughly explored the effects of gloss on camouflage, and the current evidence is unclear. In a series of experiments involving both humans and birds, Franklin et al. (2022) found no difference in survival between green beetle-like targets with mirrored and diffuse surfaces. Like us, they predicted that a glossy surface appearance would enhance survival. Their conclusion that gloss does not improve camouflage is supported by the findings of our investigation. So, perhaps glossiness is not as important to camouflage as has previously been suggested. Certainly, in the sort of environment investigated in our study (mixed deciduous woodland in spring), for iridescence to be maximally effective as camouflage against avian predators, it clearly should be combined with a matt surface appearance. That many iridescent species (and indeed non-iridescent species) are glossy therefore suggests that there must be other, perhaps non-visual, benefits to a glossy surface appearance. For instance, gloss is very often the result of surface structures that maximize hydrophobicity (Franklin and Ospina-Rozo 2021), offering protection through water repellence (Wang et al. 2020). Alternatively, glossy iridescence could protect animals in other, non-camouflaging ways, such as either a deceptive (Kjernsmo et al. 2022) or honest (Waldron et al. 2017) form of warning signal. Equally, it could simply be that there are not many large costs associated with a glossy surface.

Background gloss had the opposite effect from target gloss: all targets survived significantly better on glossier leaves. Since camouflage works by reducing the SNR (Merilaita et al. 2017; Galloway et al. 2020), increased background noise through gloss may be sufficient to provide targets with some degree of concealment irrespective of their own spectral or visual characteristics. That said, the visual complexity of the background is most effective in impeding detection when the signal lies within the distribution of the noise (Xiao and Cuthill 2016; Rowe et al. 2021), so we would predict that glossy targets would benefit more from being viewed against glossy leaves than matt targets. Interestingly, while matt targets survived significantly better than glossy targets overall, although not statistically significant (*P* = 0.054), the mortality of gloss targets tended to be greater on matt than on glossy leaves. The fact that background gloss influences the success of concealment has important implications for camouflage. Many species show a preference for backgrounds that benefit their camouflage (e.g., Kang et al. 2012, 2013a,b; Stevens et al. 2017). Perhaps some species may select backgrounds that maximize the amount of gloss-induced noise. Consideration should also be given to the fact that background glossiness may vary both spatially and temporally, with factors such as weather (in particular, rainfall) or herbivory (by leaf miners or iridescent insects themselves, for instance) potentially influencing the perceived level of specularity across a surface.

Iridescent signals are strongly dependent on illumination conditions, with directional, as opposed to diffuse, lighting maximizing the iridescent effect (Stuart-Fox et al. 2021). In addition, different habitats tend to be associated with different light environments (Endler 1993), and this might limit the range of hues that are even perceptible across an iridescent surface. Since the changeability of color seems to be the most important factor in iridescent camouflage, light environments could have important implications for the effectiveness of concealment. Understanding how different illumination conditions influence iridescence-as-camouflage would provide a valuable insight into the constraints on iridescence for concealment. Gloss is also likely to be influenced by illumination conditions. We have demonstrated that when combined with iridescent coloration, a matt surface appearance provides the greatest benefit to concealment. However, there might be situations where the benefits of a matt surface are insignificant and potentially even costly. We have alluded to the fact that many iridescent species are glossy, and we have suggested that this may be due to the non-visual benefits of the surface structure that produces a glossy appearance. But, our conclusions only really hold true for the environment in which we performed our investigation. There may be situations in other environments where gloss has visual benefits, and these should be explored. Above all, the influence of the background—color, spatial structure, and complexity as well as specularity—on the effectiveness of iridescence as camouflage should be investigated in detail.

## Supplementary Material

arad050_suppl_Supplementary_Material

## Data Availability

Analyses reported in this article can be reproduced using the data provided by Thomas et al. (2022).
