# Supplementary material for: Interactions between color and gloss in iridescent camouflage
Source: Behav Ecol. 2023 Jun 14;34(5):751–8. doi: 10.1093/beheco/arad050 (PMC10516679; doi:10.1093/beheco/arad050)
Supplement: arad050_suppl_Supplementary_Material [file arad050_suppl_supplementary_material.pdf]

Supplementary Material

The methods accompanying Figure S1 can be found in the main text of the paper. The figure plots the means of each target, based on 5 replicate measurements. The gloss measurements were repeatable (intra-class correlation = 0.86), but largely because of the substantial difference between the two gloss treatments; measurement error was high (the targets have a rounded surface, but the glossmeter's probe is flat). A variance components analysis was carried out using the VCA package in R (Schuetzenmeister & Dufey, 2022), based on a nested random effects model, with the factors gloss (two levels), colour (five levels) and target within gloss-colour treatment (10 levels). Of the total variation in gloss units, 56.0% is attributable to the two-level factor gloss, but only 2.5% to the colour variation within gloss categories and 6.1% to the individual target variation within the latter; the remaining 35.4% is measurement error.

To get a rough idea of the difference in color between the different target treatments and the leaves to which they were attached in the field, we also analyzed photographs of a subset of the targets, and all the leaves, taken at the same time as the gloss measurements (see main text). This is not an accurate assessment of target-background difference because these were single photographs, roughly normal to the target plane, and so do not capture the angle-dependent variation in color that defines iridescence. A comprehensive analysis of target reflectance, and so contrast with the background, would require measurement at multiple viewing angles and axial orientations of the target (the bidirectional reflectance distribution function; He et al., 1991).

All photographs were taken using a Nikon D80 DSLR camera (Nikon Corporation, Japan) and contained an X-Rite ColorChecker Passport (X-Rite, Grand Rapids, Michigan, USA) so that the photographs could be calibrated. All photographs were taken in plan view,

under diffuse natural lighting. Photograph calibrations (linearization and white-point balancing) were performed using a custom program written by I. C. Cuthill in MATLAB, following Stevens et al. (2007), using the RGB color values of the grey ColorChecker squares. The ‘tiff’ package in R v4.0.0 was used to read in photographs (Urbanek 2013a; R Core Team 2020) and R’s locator() function was used to outline the target and an adjacent area of leaf. Twenty targets of each treatment were used and, in order to get a representative sample of the colors (particularly important for the iridescent and static rainbow treatments, which were multicolored), 25 random pixels were selected, using R’s runif() function for x and y coordinates, within the selected areas.

RGB data were mapped to an avian color space based on blue tit (*Cyanistes caeruleus*) cone spectral sensitivities (Hart et al. 2000), these being typical of the woodland passerines seen in Leigh Woods. The latter include, besides blue tit, great tit (*Parus major*), European robin (*Erithacus rubecula*), wren (*Trogolodytes trogolodytes*), chaffinch (*Fringilla coelebs*), dunnock (*Prunella modularis*), long-tailed tit (*Aegithalos caudatus*), chiffchaff (*Phylloscopus collybita*, towards the end of the study period) and, near the woodland’s edge, house sparrow (*Passer domesticus*). The avian color space did not include the ultraviolet cone’s calculated photon catches because neither the ivy backgrounds nor the targets reflected UV (the varnish used blocked any such reflections; Kjernsmo et al. 2020).

Maxwell triangles, created using the R package ‘ternary’ (Smith 2017), represent the colors in an avian color space as the proportion of catches by each of the short (S), medium (M) and longwave (L) sensitive cone types (Figure S2a; for comparison, plots in an equivalent human color space are provided; Figure S2b). Although there is some overlap, the general pattern is that most background colors fall in a different area of color space from target colors; the colors of targets and backgrounds should be easily

discriminable and, subjectively, for humans they are (pers. obs.). The same pattern of differentiation is seen when comparing the luminance of targets and backgrounds, where targets are lighter than leaves in both avian (Figure S3a) and human (Figure S3b) vision. Note that areas of specular reflection were not included in these measurements.

REFERENCES

Barnett JB, Michalis C, Scott-Samuel NE, Cuthill IC, 2018, Distance-dependent defensive coloration in the poison frog *Dendrobates tinctorius*, Dendrobatidae. PNAS. 115:6416-6421.

CIE, 1976. CIE Colorimetry - Part 4: 1976 L*a*b* Colour Space. Joint ISO/CIE Standard. ISO 11664-4:2008(E)/CIE S 014-4/E:2007. (Vienna, Austria, Commission Internationale de l'Eclairage).

He X, Torrance K, Sillion F, Greenberg D, 1991, A comprehensive physical model for light reflection", Proc ACM SIGGRAPH '91. 175-186.

Lantz B, 2013. Machine Learning with R. Birmingham, UK: Packt Publishing Ltd.

Meyer D, Dimitriadou E, Hornik K, Weingessel A, Leisch F, 2019. e1071: Misc Functions of the Department of Statistics, Probability Theory Group (Formerly: E1071), TU Wien. R package version 1.7-3. https://CRAN.R-project.org/.

Schuetzenmeister A, Dufey F, 2022. _VCA: Variance Component Analysis_. R package version 1.4.5, <https://CRAN.R-project.org/package=VCA>.

Smith MR, 2017. Ternary: An R Package for Creating Ternary Plots, https://ms609.github.io/Ternary.

Figure S1. Comparison of glossiness (in gloss units) of glossy and matt beetle-like targets of the different color treatments, and English ivy (*Hedera helix*) leaves. Thick lines represent the median value; boxes represent upper and lower quartiles (inter-quartile range); whiskers represent maximum and minimum values. Outliers are presented as hollow circles. The upper and lower quartiles of the leaf gloss data have been extended, with green dotted lines, to aid comparison to the target gloss values.

Figure S2. Maxwell triangles for all color/gloss treatment combinations. These show how the colors of beetle-like targets (colored dots) and English ivy (*Hedera helix*) leaves (green shaded area) map into avian (a) and human (b) color space. The three axes show the percentage of photon capture by short-wave (S), medium-wave (M) and long-wave (L) cone cells. In each plot the green shaded area is a minimum convex polygon around the leaf sample colors. 'Irid' = iridescent; 'static' = 'static rainbow'. The apparently large overlap between some targets (e.g. green) and leaves in color space is rather misleading, because the leaves are represented by minimum convex polygons -- i.e. they include every single point. The densely occupied part of the color space (avian or human) is very different from the leaves for all targets.

Figure S3. Luminance of beetle-like targets and English ivy (*Hedera helix*) leaves in both avian (a) and human (b) color vision. Luminance in avian vision is plotted as the double cone photon catch; luminance in human vision is plotted as lightness in the L*a*b* color space (CIE 1976). Thick lines represent the median value; boxes represent upper and lower quartiles (inter-quartile range); whiskers represent maximum and minimum values. Outliers are presented as hollow circles. 'Irid' = iridescent; 'Stat' = 'static rainbow'; 'Gre' = green; 'Blu' = blue; 'Bla' = Black.

S1

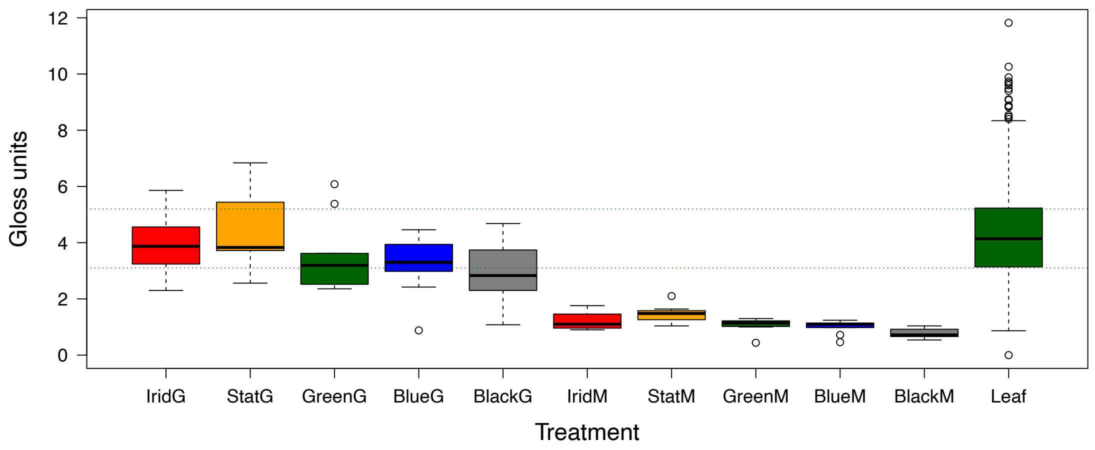

Gloss units
0
2
4
6
8
10
12
IridG
StatG
GreenG
BlueG
BlackG
IridM
StatM
GreenM
BlueM
BlackM
Leaf
Treatment

S2

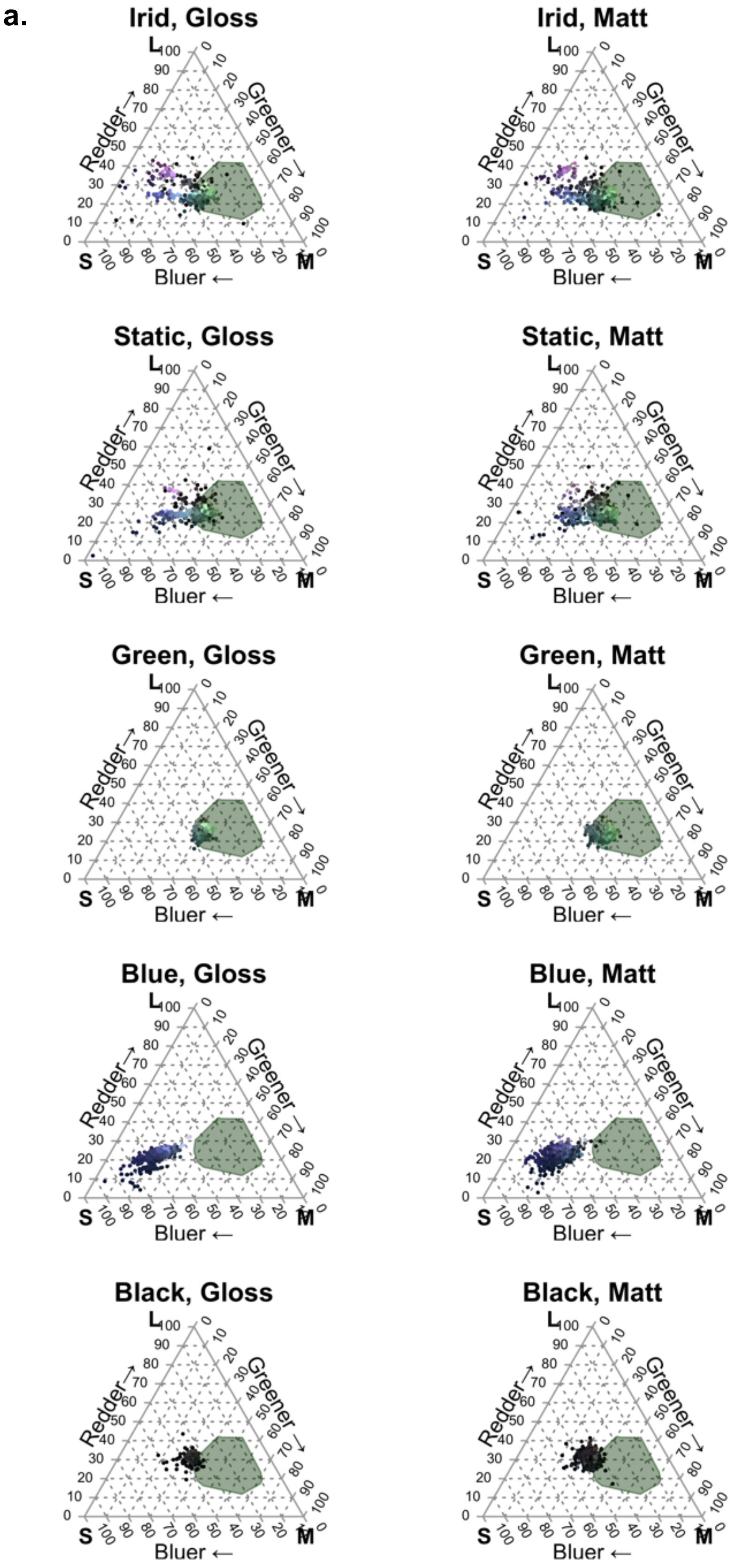
a.
Irid, Gloss
Irid, Matt
Static, Gloss
Static, Matt
Green, Gloss
Green, Matt
Blue, Gloss
Blue, Matt
Black, Gloss
Black, Matt
L
S
M
Redder →
Greener ↓
Bluer ←
0 10 20 30 40 50 60 70 80 90 100

S2

b.

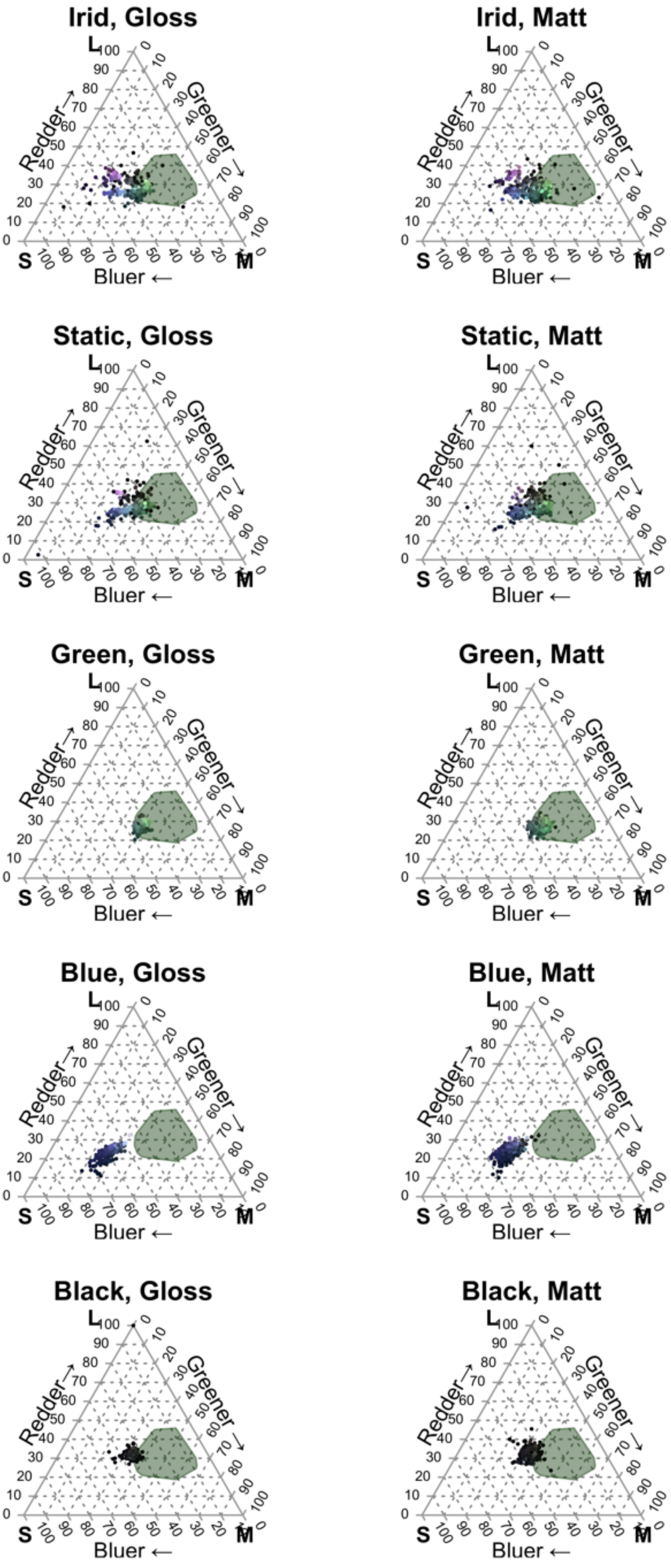

Irid, Gloss
Irid, Matt
Static, Gloss
Static, Matt
Green, Gloss
Green, Matt
Blue, Gloss
Blue, Matt
Black, Gloss
Black, Matt
L
S
M
Redder →
Greener ↓
Bluer ←

S3

a.

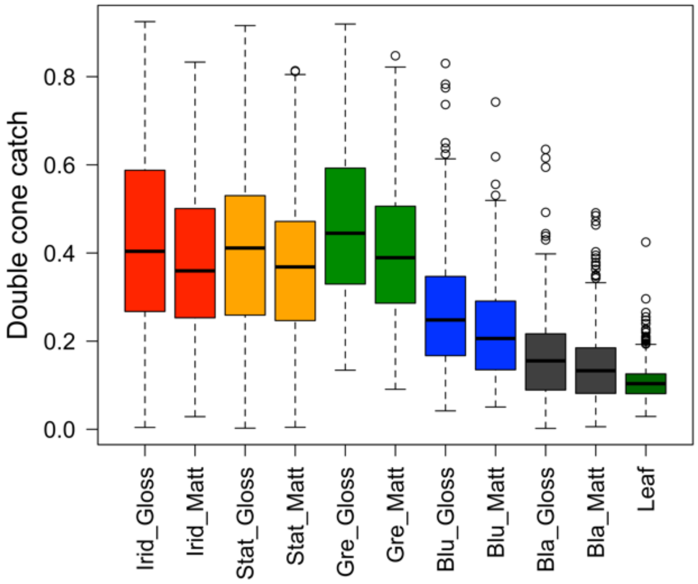
Double cone catch
0.0
0.2
0.4
0.6
0.8
Irid_Gloss
Irid_Matt
Stat_Gloss
Stat_Matt
Gre_Gloss
Gre_Matt
Blu_Gloss
Blu_Matt
Bla_Gloss
Bla_Matt
Leaf

b.

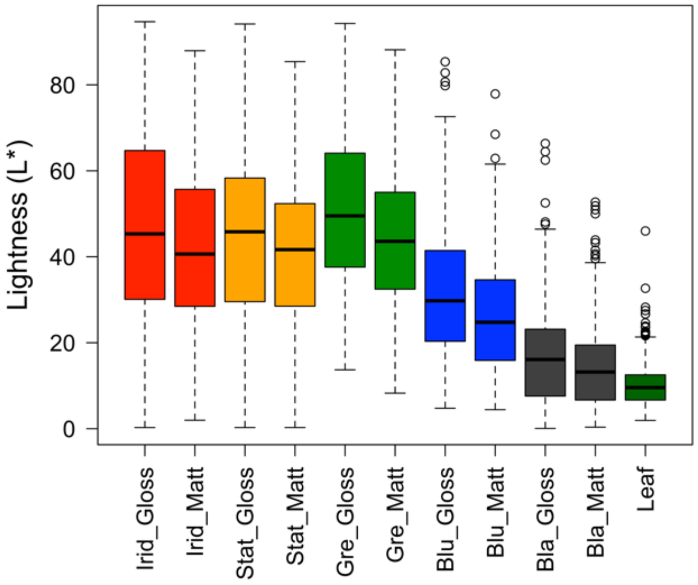
Lightness (L*)
0
20
40
60
80
Irid_Gloss
Irid_Matt
Stat_Gloss
Stat_Matt
Gre_Gloss
Gre_Matt
Blu_Gloss
Blu_Matt
Bla_Gloss
Bla_Matt
Leaf
